# Role of Cardiac Troponins in Predicting Adverse Outcomes in Acute Coronary Syndrome With Renal Dysfunction

**DOI:** 10.7759/cureus.47104

**Published:** 2023-10-16

**Authors:** Hafseena Noorayingarath, Binay K Panjiyar, Isha Gela, Lokeswaran Ramalingam

**Affiliations:** 1 Internal Medicine, Kanachur Institute of Medical Sciences, Deralakatte, IND; 2 Internal Medicine, Harvard Medical School, Boston, USA; 3 Internal Medicine, California Institute of Behavioral Neurosciences and Psychology, Fairfield, USA; 4 Internal Medicine, David Tvildiani Medical University, Tbilisi, GEO; 5 Internal Medicine, Crimean Federal University, Simferopol, RUS

**Keywords:** end-stage renal disease (esrd), acute myocardial infarction, chronic kidney disease (ckd), adverse outcome, acs (acute coronary syndrome), cardiac troponins

## Abstract

A substantial global cause of mortality as well as disability is acute myocardial infarction (AMI). It is also widespread knowledge that patients with chronic kidney disease (CKD) possess greater mortality and cardiovascular disease risks than the rest of the population. A vital biomarker for the diagnosis of AMI is high-sensitive cardiac troponin T (hs-cTnT). Individuals afflicted with severe CKD frequently exhibit increased hs-cTnT levels, which can pose a significant diagnostic challenge in cases of non-ST elevation acute coronary syndrome (NSTE-ACS) necessitating revascularization. Alteration in kidney function exerts an impact on troponin levels, making a single value less useful. As the renal population has an increased risk of non-ST-segment elevation myocardial infarction (NSTEMI), serial tracking of cardiac biomarkers is essential to detect ACS in this population. Numerous studies using algorithmic remedies based on admission troponin and spontaneous variations in troponin concentration have been put forth by researchers to address these issues. A considerable majority of CKD patients can be accurately diagnosed or excluded from having AMI using the approach, which involves serial measures. Patients who suffer from kidney impairment exhibit lesser chances of undergoing angiography or revascularization and receiving preventative therapies. Furthermore, their outcomes are comparatively poorer when compared to patients who possess normal kidney function. Despite studies indicating a higher risk of poor outcomes after AMI in this population, these patients are less likely to receive guideline-indicated care. In this study, we employed a systematic literature review (SLR) methodology to provide an account of the available studies and to draw attention to the importance of cardiac troponins in predicting unfavorable outcomes and algorithms in the prediction, diagnosis, and prognosis of patients with ACS and renal impairment. Eight papers were chosen for in-depth analysis after reviewing 86 articles from trusted publications between 2013 and August 3, 2023. The analysis considered factors such as sensitivity, severity of renal damage, algorithms used, the benefits of algorithms, and the challenges. One must examine the change in cardiac troponin (cTn) and take higher cut-off values into consideration in order to increase the sensitivity and specificity for the diagnosis of AMI. Higher levels of cTn have also been correlated prognostically to unfavorable outcomes like incident heart failure and death from cardiovascular causes. Also, raised troponin levels have been linked to all-cause and cardiovascular death in both dialysis patients and patients with CKD who did not receive dialysis. Future studies should concentrate on whether troponin testing can reclassify risk and provide treatment in people with CKD who are at the greatest threat of death. The clinical practice benefits of routinely measuring cardiac troponin concentrations are largely unknown. Future research should also concentrate on figuring out how troponin testing can influence clinical management and how to address the root reasons for chronic hs-cTnT elevation in patients with CKD, which may include elements like uremic toxicity, macrovascular or microvascular ischemia, anemia, as well as reduced renal clearance.

## Introduction and background

Patients with renal impairment have a higher risk of myocardial infarction (MI), which can occur without ST-segment elevation in the electrocardiogram (ECG) and present with uncommon symptoms [1]. The diagnosis and treatment of acute coronary syndrome (ACS) have undergone a revolution as a result of the emergence of increasingly sensitive and specific serum cardiac biomarkers [2]. The assessment of troponins in patients with acute kidney injury (AKI), chronic kidney disease (CKD), and end-stage kidney disease (ESKD), despite the fact that the troponins currently in use are thought to be highly sensitive cardiac biomarkers in the general population, has long been a source of diagnostic unreliability [2]. Previous studies have reported higher cutoffs for high-sensitivity cardiac troponin T (hs-cTnT) levels in patients with CKD, but these cutoffs had lower specificity and overall accuracy compared to the healthy population [3].

Cardiac troponin T (cTnT), a vital biomarker in the detection of acute myocardial infarction (AMI), is a low-molecular-weight protein that belongs to the troponin complex and functions as a crucial element of the myofibrillar contractile apparatus [4]. Release of cardiac troponins into circulation due to loss of integrity of cardiac myocyte membranes can be determined using highly sensitive cTnT assays to identify AMI [4]. Patients with AMI and ESKD have significantly different cTnT form compositions, especially within the time window of the clinical diagnosis of AMI (12 h following the beginning of symptoms) [5]. Rapid changes to kidney function and renal clearance alter the metabolism and excretion of troponins [2]. Such alterations can create uncertainty around the interpretation of troponin levels, as they could be affected more by the kidney injury itself rather than the presence or severity of ACS. Troponin levels have been investigated as independent predictors of morbidity and mortality after an acute ischemic event, but their prognostic significance in CKD patients experiencing ACS remains unclear [6]. There is also uncertainty about how elevated troponin levels affect the effectiveness of interventions or treatment strategies [6].

We tried to systematically review research data regarding the diagnostic accuracy of raised troponin levels for ACS monitoring in patients with CKD, the extent to which raised troponin level impacts the comparative effectiveness of interventions or treatment approaches for ACS in this population, and the prognostic role of elevated troponin levels in figuring out short- and long-term outcomes in patients with CKD and suspected ACS.

## Review

Methods

**Figure 1:**
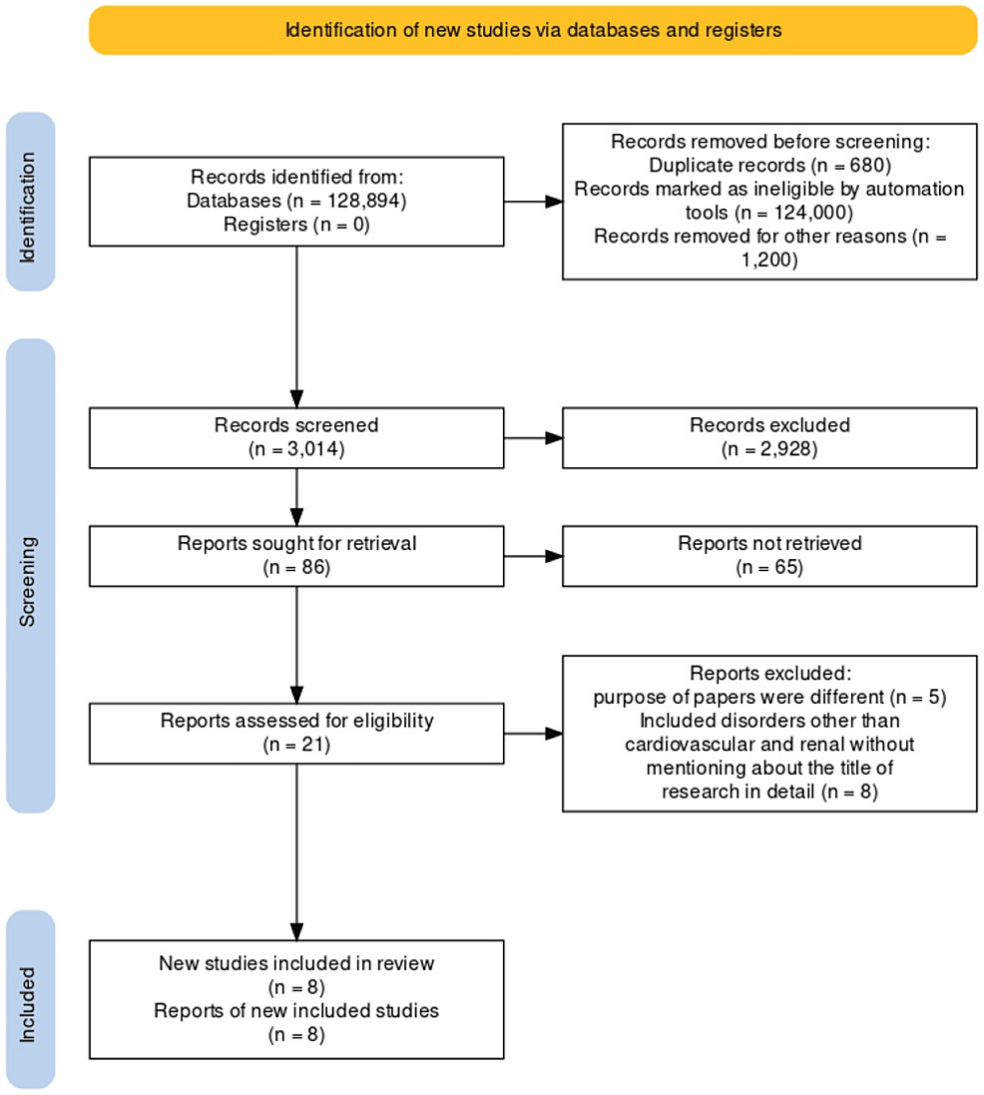
PRISMA flow diagram illustrating the search strategy and study selection process for the systematic review. PRISMA: Preferred Reporting Items for Systematic Reviews and Meta-Analyses

In this review, we specifically address the relevance of cardiac troponins in the prediction of unfavorable outcomes in ACS with renal failure. Animal studies and studies involving clinical information other than ACS and renal impairment were excluded. Only data extracted from published papers are used in the review, which complies with the Preferred Reporting Items for Systematic Reviews and Meta-Analyses (PRISMA) for 2020 guidelines in Figure 1 and does not need ethical approval [7]. 

Systematic literature search and study selection

We searched extensively for appropriate publications by using PubMed, including Medline and Google Scholar. On PubMed, we looked for studies that had been referenced in review articles, editorials, and commentaries. But we also continued looking for further research that adhered to our inclusion standards. We independently assessed the collection of abstracts for inclusion following defined criteria. A well-defined clinical cohort and the application of cardiac troponins in the study were the main criteria. Animal studies and reviews with narratives were not included. Unanimity was attained through discussion upon an independent review by four reviewers.

Inclusion and exclusion criteria

To accomplish our study's aims we established specific inclusion and exclusion standards. Table 1 consists of a summary of our criteria.

**Table 1:** Indicating the criteria used for the literature search.

Inclusion Criteria	Exclusion Criteria
Human studies	Animal studies
Published articles in the last 10 years (2013 to 2023)	Narrative reviews
Gender: All	Articles in languages other than English
Adults: >19 years of age	Age: <19 years of age
Published articles with PubMed indexing and articles from Google Scholar	Studies involving clinical data other than acute coronary syndrome and renal impairment
Article type: systematic reviews, meta-analysis, randomized controlled trials, clinical trials	Papers that needed to be purchased
Articles in English language	
Free papers	

Search strategy

We carried out a thorough analysis of the literature applying the population, intervention/condition, control/comparison, and outcome (PICO) criteria. Using appropriate keywords like cardiac troponins, adverse outcomes, ACS, ESRD, AMI, and CKD, the search was done on databases like PubMed (including Medline) and Google Scholar libraries. A detailed search plan was devised, utilizing the medical subject heading (MeSH) approach for Google Scholar and PubMed (including Medline), as demonstrated in Table 2.

**Table 2:** Displaying the search strategy, the search engines utilized, and the number of results.

Data Base	Search Strategy	Results
PubMed	cardiac troponins OR acute coronary syndrome OR STEMI OR NSTEMI OR unstable angina OR kidney failure OR kidney dysfunction AND "2013/01/01": "2023/03/08"	105794
Google Scholar	acute coronary syndrome OR heart attack OR STEMI OR NSTEMI OR unstable angina OR kidney dysfunction OR kidney failure AND cardiac troponins	23100

Quality appraisal

We made use of a number of quality assessment tools in order to ensure the reliability of the articles we chose. For systematic reviews and meta-analyses, we relied on the PRISMA checklist and the Cochrane bias tool assessment for randomized clinical trials. The Newcastle-Ottawa tool scale was employed to evaluate non-randomized clinical trials. Using the Critical Appraisal Skills Program (CASP) checklist, we analyzed the validity of qualitative studies, as can be seen in Table 3. We assessed the article's quality using the Scale for the Assessment of Narrative Review Articles (SANRA) in order to eliminate any ambiguity in the classification.

**Table 3:** Displaying the application of quality appraisal tools. PRISMA, Preferred reporting items for systematic reviews and meta-analyses; SANRA, Scale for the evaluation of non-systematic review articles; RCT, randomized control trials

Quality Appraisal Tools Used	Type of Study
Cochrane bias tool assessment	RCT
PRISMA checklist	Non-RCT and observational studies
The Newcastle-Ottawa tool scale	Systematic reviews
SANRA	Any other without a clear method section

Results

We acquired 128894 articles after carrying out searches in three specific databases: PubMed, Medline, and Google Scholar. After meticulously reviewing each paper, we utilized a variety of criteria to discard 125880 articles. We omitted 2928 of the remaining 3014 papers given that their titles or abstracts were duplicates or unsuitable for use. After carefully going through the remaining 86 papers, we cut out yet another 78 because the information they contained did not meet our inclusion requirements. The final phase was an extensive quality assurance of the eight papers that remained and all of which fulfilled our standards. Our final systematic review consists of these eight articles. Each one is fully outlined in Table 4.

**Table 4:** Summary of the results of the selected papers. Hs-cTnT, high-sensitive cardiac troponin T; Hs-cTnI, high-sensitive cardiac troponin I; NSTE-AMI, non-ST-elevated acute myocardial infarction; STE-AMI, ST-elevated acute myocardial infarction; BNP, brain natriuretic peptide; eGFR, estimated glomerular filtration rate

Author/Year	Country	Study Design	Data Base Used	Conclusion
Alushi et al., 2021 [3]	Germany	Observational study	Data from a local MI registry which included patients from three tertiary cardiovascular centers.	Hs-cTnT has greater diagnostic performance in patients with severe CKD and suspected ACS when using higher assay-specific cutoff levels and early absolute changes.
Kraus et al., 2018 [1]	Germany	Cohort study	Data sources utilized include a prospective cohort study, a clinical registry, and a Clinical Data Warehouse.	The diagnostic accuracy of high-sensitivity cardiac troponins in patients with CKD with suspected NSTE-AMI has been improved by an algorithm based on admission troponin and rapid changes in troponin level.
Miller-Hodges et al., 2018 [8]	UK	Editorial	Data from the electronic patient record using TrakCare were used.	Patients with renal impairment are identified as being at high risk by high-sensitivity cardiac troponin, but this test's lower specificity for type 1 myocardial infarction raises the chance of further evaluation and therapy.
Yang et al., 2017 [9]	China	Observational study	Inpatient databases of a hospital from September 2010 to June 2014 were used.	The type and severity of AMI, BNP, and eGFR are all correlated with hs-TnT levels. In CKD patients, 129.45 ng/L is the ideal cutoff value for the diagnosis of AMI.
Stacy et al., 2014 [6]	USA	Systematic review	Medline, Embase, and the Cochrane Central Register of Controlled Trials	Although varying sensitivity and specificity estimates limit the diagnostic utility of troponin levels, they can be used to identify patients with a poor prognosis for CKD and suspected ACS.
Gallacher et al., 2022 [10]	UK	Randomized controlled trial	Enrolled a total of 48282 patients across 10 secondary and tertiary hospitals into the study.	Kidney impairment patients have higher cardiac troponin concentrations and increased diagnosis of type 1 myocardial infarction, but they are less likely to undergo angiography, revascularization, or preventative therapies.
Michos et al., 2014 [11]	USA	Systematic review & Meta-analysis	They searched through Medline, Embase, and the Cochrane Central Register of Controlled Trials. Stata/IC, version 12.1 (StataCorp), was used for all meta-analyses.	Elevated troponin levels in CKD patients without suspected ACS have a worse prognosis, highlighting the need for future studies to determine its suitability for risk classification.
Gunsolus et al., 2018 [12]	USA	Observational study	Data from a specific cohort of patients presenting at a medical center through the emergency department.	Renal impairment and dialysis lowered hs-cTnI's ability to rule in MI while raising all-cause mortality in patients with higher concentrations. Though hs-cTnI might rule out MI, clinicians should be mindful of its impact.

Discussions

Hs-cTnI and hs-cTnT tests are growing in popularity in clinical laboratories across the world. Analytically, high-sensitivity assays have been found to be outstanding, minimizing the number of false positive and false negative results [12]. Due to this, early rule-in and rule-out techniques for MI have been developed, cutting the time needed for a diagnosis from six to three hours [12]. Patients with CKD often face cardiovascular risks, which increase with renal impairment [13]. Researchers have investigated the diagnostic correctness of hs-cTnT for the diagnosis of non-ST elevation acute coronary syndrome (NSTE-ACS) needing revascularization in a sizable cohort of patients with severe CKD (eGFR <30 mL/min/1.73 m^2^), involving individuals with CKD G5D [3]. When compared to patients who were not revascularized, these patients' median hs-cTnT values were considerably greater, which implies that regardless of baseline hs-cTnT levels, an apparent increase in hs-cTnT levels can be observed in the presence of NSTE-ACS [3]. Uncertainty encircles the reason for this chronic elevation that is being researched.

Studies reveal that diagnostic accuracy at presentation was high in patients with renal dysfunction, with an AUC ranging from 0.87 to 0.89 [14]. Patients with renal dysfunction had ideal cutoff levels that were two to three times higher than those with normal renal function [14]. In patients with renal dysfunction, cTn additionally maintained its prognostic value and predicted two-year survival [14]. These findings are in line with a study conducted by Stacy SR and colleagues [6]. Furthermore, the diagnostic accuracy increased with serial sampling [3].

According to a study by Kraus et al., using 2.5-fold changes in hs-cTnT levels at three hours, two hs-cTnT assays possess better diagnostic accuracy [1]. Dialysis patients were not included, and patients with severe CKD were underrepresented. Contrary to patients with normal renal function, the study found that patients with severe CKD had reduced sensitivity and specificity. A study by Miller-Hodges et al. also showed that patients with renal impairment had poorer sensitivity and negative predictive values for the main outcome of MI or cardiac mortality after 30 days and in such individuals, the application of diagnostic thresholds exceeding the 99th percentile might boost specificity for type 1 MI [8].

Greater ROC-derived cutoff levels for patients undergoing dialysis were obtained, which is consistent with the Yang et al. survey [3,9]. The cutoff value was lowest in CKD G3 patients and highest in dialysis patients, but the assessment proved complicated due to the lack of a control cohort [3,9]. Recent research indicates a strong correlation between hs-cTnT and major adverse cardiovascular events and all-cause mortality [14]. Dialysis is a standalone predictor of cardiovascular and all-cause mortality [4,14]. Patients with renal disease have been demonstrated to have elevated levels of cTnI and cTnT despite no evidence of ACS, including MI [12]. Studies have suggested a data-driven choice-making algorithm for serial measurements in CKD patients with a focus on troponin levels [1]. When using hs-cTnI, the algorithm has a high diagnostic accuracy rate compared to using the hs-cTnT assay [1,12]. For the remaining, additional observation and diagnostic testing are required [1].

The study by Gunsolus et al. [12] draws attention to the finding that, while the assay's sensitivity and NPV exhibited no appreciable influence on renal function, the clinical specificity and PPV for MI moved downward with declining renal function [12]. The study additionally showed that renal impairment reduced the diagnostic efficacy of both cTnT and cTnI tests. Patients with CKD and suspected NSTE-AMI, however, are complex, high-risk patients, and a decision must weigh each person's risk and benefit [1]. According to the analysis of the study results by Gallacher et al., patients with kidney impairment may be more likely to experience type 2 MI or nonischemic myocardial injury, which could account for the reason why outcomes did not improve after the incorporation of high-sensitivity cardiac troponin testing [10]. To comprehend the mechanism beneath the link between raised troponin levels and unfavorable outcomes, more research is required [11]. A high troponin level could be just a sign of underlying CAD and the risk it carries [11]. The question of whether troponin measurement gives value over known clinical covariates is vital, even though it reflects injury rather than exposure may be confounding [11]. 

To ensure precise hs-cTnT measurements, the assay's calibration and accuracy must be monitored [5]. Despite not being identical to the cTnT forms found in the sera of patients with ESRD and AMI, the quality control materials used for the hs-cTnT assay possess considerable diagnostic and prognostic value [5]. Furthermore, regardless of the degree of kidney impairment, coronary revascularization and preventative treatments were linked to a reduced risk of the primary outcome in patients with type 1 MI, in line with prior studies [10]. Additionally, there is a shortage of solid data supporting the use of such treatments in kidney impairment patients, who have the highest cardiovascular risk, and this should be preferred for future studies.

Limitations

We have constraints with our literature review. We especially targeted those at least 19 years old, narrowed our research to English articles published during the last ten years, and utilized just free articles. The review included studies with a variety of designs, which may have limited the range to which the results could be generalized. The possible impact of confounding variables, which include medication intake or comorbidities, on the association between kidney damage and cardiac incidents was not reviewed in the study. For precise findings, more studies are required.

## Conclusions

The significance of CKD among patients with MI has been highlighted in reports over the past ten years. According to findings from this study, individuals with CKD continue to receive less evidence-based treatments and die at significantly greater rates. Higher accuracy in diagnosing is preserved in patients with suspected AMI and renal impairment by more sensitive cTn testing. Assay-specific recommended cutoff levels, which are greater in patients with renal impairment, should be taken into consideration along with early absolute changes to enable the most beneficial and effective clinical use. So far, modified algorithms for the analysis of troponin in renal patients have had, at best, limited impact. It is logical to continue serial troponin administration in patients with kidney illness. For a quicker diagnosis and improved outcomes following ACS, further research is required to find new biomarkers and to better utilize the ones that are now available in patients with AKI, CKD, and ESKD. 

Levels of troponin can help distinguish individuals with a poor prognosis in those with CKD and suspected ACS, although their diagnostic accuracy is constrained by various estimations of specificity and sensitivity. According to the severity of CKD, the relative prognostic significance of cTn concentrations varies, and there may be differences in how much prognostically harmful and less harmful entities contribute in patients with and without ACS. When evaluating cTn concentrations in patients admitted with suspected ACS, it is important to take these interrelations into account. Regardless of this, individuals with kidney damage continued to have poorer results than those with regular kidney function and were not any more likely to undergo angiography, revascularization, or prophylactic treatments. Evaluating medication regimens that use troponin tests in their algorithms should be the main objective of new studies, especially for patients who are more at risk.
